# Epigenetic Strategies to Boost Cancer Immunotherapies

**DOI:** 10.3390/ijms18061108

**Published:** 2017-05-23

**Authors:** Maria J. Barrero

**Affiliations:** CNIO-Lilly Epigenetics Laboratory, Spanish National Cancer Research Center (CNIO), C/Melchor Fernández Almagro, 3. 28029 Madrid, Spain; mjbarrero@cnio.es; Tel.: +34-917-328-000

**Keywords:** immunotherapy, cancer, epigenetics

## Abstract

Recently, immunotherapeutic approaches have shown impressive responses in a subset of cancer patients. However, the rate of success is low and a large percentage of treated patients do not experience clinical benefits. Therefore, additional strategies are needed to improve responses and select responsive patients. Emerging data suggest that epigenetic drugs can improve the responses to immunotherapy. Understanding the mechanisms of resistance to immunotherapy and the epigenetic events that take place during immune evasion is critical to providing a rational combined use of immunotherapies and epigenetic drugs. This review focuses in the epigenetic mechanisms involved in the responses to immunotherapy and how current drugs that target epigenetic regulators impact on them.

## 1. Introduction

Back in 1957 Burnet and Thomas proposed the theory of cancer immunosurveillance suggesting that the immune system has the capacity to detect and destroy abnormal cells and may prevent the growth of tumors. Cancer cells express cancer antigens, mainly produced by the accumulation of mutations or re-expression of embryonic genes, which can be recognized by the adaptive immune system. However, this strategy is not always effective in the eradication of cancer cells. Low mutational rates or improper antigen presentation might result in the generation of insufficient functional tumor antigen-specific T cells. There is also the possibility that tumor antigen–specific T cells are generated but fail to infiltrate into the tumor parenchyma. In addition, a large number of immunosuppressive mechanisms that typically operate within the tumor microenvironment can attenuate the immune response. These immunosuppressive mechanisms are, in general, natural responses to prevent autoimmunity during chronic antigen exposure. Eventually, cancer cells might also develop mechanisms of resistance to the persistent action of the immune system. Cancer immunotherapies are focused in boosting preexisting immune responses, such as immune checkpoint-oriented therapies, or in the generation new immunological responses against tumors.

The recent success of immunotherapy in a subset of cancer patients suggest that the immune system offers a feasible opportunity for therapeutic intervention. Unfortunately, despite showing impressive responses in a percentage of cancer patients, more than half of the treated patients do not experience clinical benefits. Thus, new strategies to improve the efficacy of these approaches are needed. Emerging data suggest that epigenetic drugs can boost cancer patient’s responses to immunotherapeutic treatments. In addition, understanding the role of chromatin in the regulation of immune-related pathways might serve as a basis for the development on novel immunotherapeutic strategies.

## 2. Chromatin and Epigenetic Inhibitors

The genome is organized into chromatin structures that play critical roles in gene expression. The fundamental unit of chromatin is the nucleosome, composed of two copies each of the core histones H2A, H2B, H3, and H4, wrapped by DNA. Chromatin offers a physical barrier to the efficient recruitment and processivity of the RNA Polymerase II (Poll l) and, thus, impedes transcription [1]. The N-terminal tails of histones are relatively accessible to enzymatic modifications, such as acetylation, methylation, phosphorylation, ubiquitination, and sumoylation, and the cytosine residues of DNA can be modified by methylation and hydroxymethylation. Chromatin covalent modifications are dynamically regulated and result from an equilibrium between the action of enzymes that deposit the mark and enzymes able to remove it. These modifications can influence the degree of condensation of chromatin per se and/or facilitate the recruitment of structural or effector proteins, such as remodeling complexes, that directly affect the condensation of chromatin [2]. Usually, the level of chromatin compaction and the transcriptional status of genes is not determined by the presence of one single type of modification but by the coincident action of several modifications present at a given region of the genome. As a result of the combined action of modifications, certain areas of the genome are organized into heavily condensed chromatin structures, such as centromeric regions, that offer little room for transcriptional regulation. Other regions contain genes that are silenced, but that can be activated by certain stimuli. These mechanisms, leading to transcriptional activation or repression, are likely gene specific and highly influenced by the transcription factors bound at the regulatory regions of a particular gene at a given time. The level of compaction of the chromatin influences the accessibility of transcription factors to their responsive elements in the DNA and transcription factors, in turn, have the ability to recruit chromatin-modifying enzymes that affect the structure of the chromatin. Therefore, the interaction between transcription factors and chromatin structure is complex.

Over the last decades, highly potent and specific inhibitors of chromatin-modifying enzymes, chromatin-related complexes, and proteins able to recognize specific histone marks have been developed. Some of these inhibitors have shown promising results in cancer treatment in clinical and/or preclinical studies.

DNA methylation is involved in gene repression and maintained by DNA methyltransferases (DNMTs). The DNMTs inhibitors azacytidine (AZA) and decitabine (DAC) are the most successful with the longest history of epigenetic drugs used in cancer treatment to date. These compounds are nucleoside analogs that are incorporated into DNA during replication and form covalent adducts with DNMTs depleting in this way the pool of active enzymes in the cell. AZA and DAC are currently approved for the treatment of myelodysplastic syndrome and acute myeloid leukemia [3]. Traditionally, it has been accepted that the therapeutic effects of DNA hypomethylating agents are mediated through the reactivation of the expression of tumor suppressors, however recent findings suggest that the mechanisms of action of these drugs are more complex.

Histone acetylation is involved in gene expression and is controlled by the action of histone acetyltransferases (HATs) and histone deacetylases (HDACs). HDAC inhibitors have been approved for the treatment of cutaneous or peripheral T cell lymphomas and multiple myeloma. HDACi have shown limited single-agent activity in other malignancies [4]. Currently, several clinical trials are ongoing to test the efficacy of both DNMTs and HDACs inhibitors both in hematologic and solid tumors, many of them in combination with standard care drugs.

Histone acetylation stimulates gene transcription through the recruitment of effector factors that, in turn, facilitate the proper recruitment and functionality of RNA polymerase II. Proteins that contain bromodomains have the potential to bind to acetylated histones in the chromatin and contribute to transcriptional activation [5]. Recently, inhibitors able to block the binding of bromodomains to acetylated histones have been developed [6]. The first reported bromodomain inhibitors (JQ1 and I-BET151) target the bromodomain of the BET family of proteins and have demonstrated antiproliferative activity in preclinical studies [7,8]. Antiproliferative effects are mainly mediated through the inhibition of the BET containing protein BRD4, that is essential for high-level expression of oncogenes that have very high levels of acetylation in their promoters [9]. Inhibitors of BET proteins have recently entered early-stage clinical trials.

In addition to BRD4, the human proteome encodes at least 45 diverse bromodomain-containing proteins [6]. These include several HATs such as CREBBP and EP300. Recently, dual inhibitors of the bromodomains of CREBBP and EP300 have been developed and preclinical studies suggest that they have therapeutic potential [10,11,12,13,14,15]. Inhibition of the CREBBP/EP300 bromodomains likely impedes the proper binding of these factors to acetylated chromatin which, in turn, prevents the acetylation of histones leading to a decrease in gene expression [12].

In addition to DNMTs, HDACs and bromodomain inhibitors, drugs that inhibit other classes of epigenetic regulators have been described, such as inhibitors of histone methyltransferases (HMTs) and demethylates (HDMTs). Inhibitors targeting the histone methyltransferases DOT1L and EZH2, as well as the demethylase KDM1 (also known as LSD1), have recently entered the first stages of clinical trials in cancer therapy and might hold the potential to modulate the cancer-immune system axis [16].

## 3. Reinvigoration of Exhausted T Cells

During chronic antigen exposure in cancer T cells become exhausted and dysfunctional [17]. As discussed above, rather than anomalous, this process seems to be a natural safeguard mechanism that impairs prolonged immune responses that may result in autoimmunity. Exhausted T cells exhibit defective proliferative capacities, reduced cytokine production and diminish lytic functions. The loss of these important properties is coincident with the upregulation of several surface receptors, including PD-1 and CTLA-4. Concomitantly, the expression of PD-1 ligands PD-L1 and PD-L2 in tumor cells is induced by cytokines and inflammatory factors, such as interferon gamma (IFNγ), mostly secreted by activated T cells infiltrated in the tumor. After ligand binding, PD-1 inhibits kinase-signaling pathways involved in T-cell activation. Accordingly, antibodies that block the interaction of CTLA-4 or PD-1 with their ligands are able to reinvigorate exhausted T cells and have shown long-term favorable responses in a percentage of cancer patients, including complete tumor regression and remission in patients with far-progressed cancers that were refractory to standard treatments [18,19,20].

**Figure 1:**
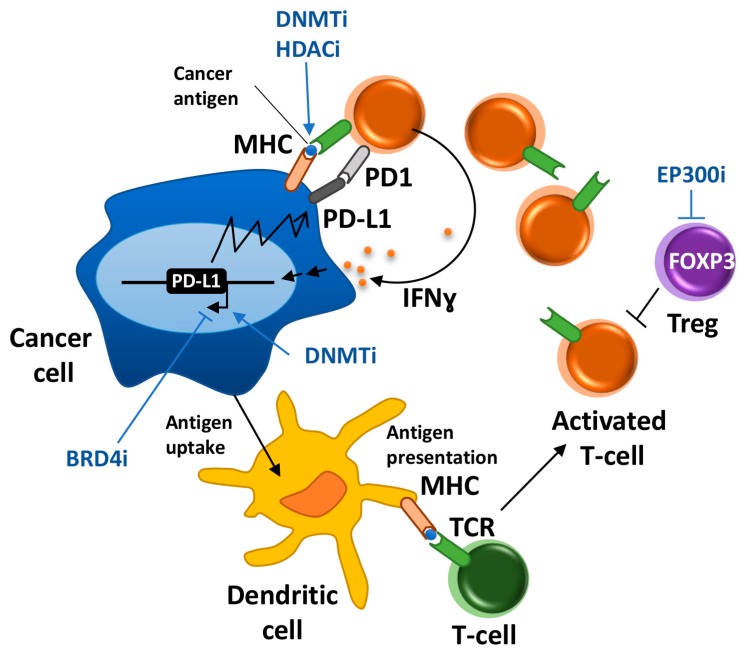
Summary of major events involved in cancer immunosurveillance and the described effects of epigenetic drugs. Cancer-specific antigens expressed in the tumor cell are uptaken and processed by dendritic cells. Mature dendritic cells migrate to the lymph node (not depicted) where they present antigens to T-cells mediating their activation. Activated T cells migrate to the tumor and recognize cancer cells that present cancer-associated antigens. Activated T cells carry out effective functions to eliminate cancer cells, among them secretion of IFNγ that will promote apoptotic effects in cancer cells. Sustained exposure to IFNγ leads the expression of PD-L1 in cancer cells and T cell exhaustion. Blue arrows indicate critical steps activated or blocked by epigenetic drugs.

Favorable responses to anti-PD-1/PD-L1 therapies typically correlate with high levels of PD-L1 expression in tumors [21,22]. There is also a strong association between low DNA methylation status of the PD-L1 promoter in tumors and poor survival of acute myeloid leukemia (AML) patients, suggesting that DNA methylation may block the induction of PD-L1 in tumors in response to IFNγ or other immunological cues [23]. Clinical trials have shown that a small percentage of NSCLC patients previously treated with the DNA hypomethylating agent AZA show improved responses to anti-PD1/PD-L1 therapies [24]. AZA treatment alone or in combination with other epigenetic inhibitors improves the therapeutic efficacy of anti-PD-L1 and anti-CTLA4 treatments in mouse models of melanoma and ovarian cancer [25,26]. A potential explanation for this outcome is the increase in PD-L1 expression caused by AZA in cancer cells (Figure 1) [27,28]. Upregulation of PD-L1, PD-L2, PD-1, and CTLA4 expression has been also detected in myelodysplastic syndromes patients treated with DNA hypomethylating agents and has been associated with the emergence of resistance to AZA [29]. Overall, these data might provide a rationale to combine DNA hypomethylating agents with PD-1/PD-L1 immune checkpoint blockade to avoid immune escape through PD-L1 upregulation after exposure to DNA hypomethylating agents. However, it should be taken into account that the effects of AZA in the expression of immunomodulatory signatures are diverse and additional aspects might be involved in boosting the anti-tumor immune responses mediated by anti-PD-L1 and anti-CTLA4 treatments, as explained below.

Similar to AZA, HDAC inhibitors upregulate the expression of PD-L1 and PD-L2 in melanoma cells providing a rationale for the reduced tumor progression and increased survival of HDAC inhibitors and PD-1 blockade combinations observed in a murine model of melanoma compared with single-agent treatments [30]. However, due to the pleiotropic effects of epigenetic regulators (Table 1), combinatorial therapies with immune checkpoints might have more complex responses than anticipated. For example, epigenetic drugs are also likely to have direct effects in the epigenome of immune cells. In this regard, the treatment with anti–PD-1 or anti–CTLA-4 antibodies plus the HDAC inhibitor entinostat has been reported to cause eradication of tumors and metastasis in a mouse model of breast cancer, mainly through inhibition of granulocytic myeloid-derived immune suppressive cells (MDSCs) [31]. Additionally, it has been proposed that HDAC inhibitors enhance the response to PD-1 blockade in multiple lung tumor models through the stimulation of T-cell chemokine expression [32]. Despite the relatively low existing understanding of the mechanisms of action of HDACs and DNMTs inhibitors in the immune system, the promising results obtained in preclinical models have favored the recent development of several clinical trials testing combinations of anti-PD-L1/PD-1 or CTLA-4 therapies and HDAC or DNMTs inhibitors for the treatment of several types of cancers (https://clinicaltrials.gov).

Eventually, epigenetic inhibitors can be used to downregulate the expression of PD-1 or PD-L1 alleviating the process of T-cell exhaustion. The bromodomain inhibitor JQ1 suppresses PD-L1 gene expression in ovarian cancer cell lines that express high levels of this gene and increases the activity of cytotoxic T cells limiting tumor progression in a mouse model of ovarian cancer (Figure 1) [33]. Importantly, JQ1 is able to revert the induction of PD-L1 mediated by IFNγ in vitro, suggesting that it could also prevent the induction of PD-L1 expression by IFNγ secreted by infiltrating T cells [33]. In addition, BRD4 inhibitors have also shown antiproliferative effects in ovarian cancer cells [34]. Therefore, JQ1 might have dual anti-tumor actions: it slows down the proliferation of tumors and prevents T cell exhaustion.

Unfortunately, despite the impressive results achieved in some patients, the majority of patients do not respond to PD-1/PD-L1 checkpoint blockade, with many patients demonstrating a transient restoration of T cell function followed by disease relapse [18]. Although checkpoint blockade temporarily restores T cell effector function, the epigenetic landscape of exhausted T cells, which significantly differs from non-exhausted T cells, is not reinvigorated with treatment and might be responsible for the reversion back to an exhausted state after withdrawal of treatment [35]. It is still unknown whether patients that respond to treatment might have exhausted T cells with a less-committed epigenetic landscape that facilitates their reinvigoration at the epigenetic level. In any case, epigenetic drugs could be used in combination with PD-1/PD-L1 checkpoint blockade to reinvigorate the epigenome of exhausted T cells and achieve durable responses.

## 4. Antigen Presentation and HLA Expression

Recognition of cancer cells by tumor-specific T cells is achieved by the presentation of antigenic peptides on major histocompatibility complex (MHC) class I molecules on the surface of cancer cells. These cancer-specific antigens might be the result of mutagenesis, cancer-restricted alternative splicing, or re-expression of embryonic antigens typically silent in healthy adult tissues. Downregulation of the expression of tumor-associated antigens and MHC class I genes constitutes a common mechanism of immune evasion. DNA methylation appears to be involved in this process, as judged by the upregulation in the expression of cancer testis antigens (CTAs), which are expressed in placenta and testis, but not in the rest of adult healthy tissues, and MHC class I genes by DNMT inhibitors in cancer cells [36,37]. AZA and HDAC inhibitors have been also described to cause the reactivation of repetitive elements such as endogenous retroviruses and transposable elements [26,38,39] which could eventually contribute to introduce mutations and subsequent production of new antigens that can be potentially targeted by the immune system. In addition, the promoters of several CTAs are enriched in the repressive chromatin mark H3K27me3 in lung cancer cell lines that express low levels of these genes. Accordingly, inhibition of EZH2, the enzyme involved in H3K27me3 deposition, potentiates the induction of CTLAs by DAC in these cell lines [40]. Additionally, knockdown of the demethylases KDM1 or KDM5B also enhanced DAC-mediated activation of these cancer–testis genes [40]. These demethylases specifically remove H3K4 methylation, a modification involved in gene activation, and although their knockdown caused markedly-increased levels of H3K4me2 at several CTAs the activation of these genes was only observed in combination with DAC. These results highlight the complexity of the interplay between histone and DNA modifications to regulate gene expression in living cells. Importantly, promising results have been recently reported for the first Phase I clinical trial testing an inhibitor of KDM1 in acute leukemia [41], and additional clinical trials for KDM1 inhibitors on sickle cell disease, small cell lung cancer, and other advanced malignancies have been recently launched (NCT02712905, NCT02913443 and NCT03132324, https://clinicaltrials.gov). Inhibitors against KDM5 that show anti-cancer properties in preclinical models have also been recently developed [42]. Testing potential effects of KDM1 and KDM5 inhibitors in boosting tumor antigenicity is granted as these inhibitors move into the clinic and more knowledge is gained in preclinical models. Finally, similarly to DNMT inhibitors, HDAC inhibitors have also been found to upregulate the expression of cancer-specific antigens and components of the tumor antigen processing and MHC presentation pathway [43].

Despite the fact that tumor cells can present antigens, they have very limited capacity to activate T cells. In fact, dendritic cells (DCs) are the antigen-presenting cells that play a pivotal role in the activation of T cells by cancer-associated antigens (Figure 1). In addition to proper antigen presentation by the MHC, T cell activation requires further stimulation by the co-stimulatory surface molecules B7 on DCs and CD28 on T cells. DCs can sample tumor antigens through the capture of dying tumor cells and phagocytosis of small quantities of membrane from live cells [44]. Therefore, it is possible that epigenetic inhibitors that have apoptotic effects on tumors will also facilitate antigen presentation by DCs and subsequent activation of T cells. Additional potential effects of epigenetic drugs in the expression of critical molecules involved in antigen presentation and stimulation of T cells by DCs remain mainly unexplored.

## 5. Adoptive Cell Transfer and Expression of Critical Tumor Neoantigens

Adoptive cell transfer (ACT) consists of purification, in vitro modification, expansion, and infusion back into patients of therapeutically-relevant immune cell types. It has been shown that adoptive cell transfer utilizing autologous tumor-infiltrating lymphocytes (TIL) mediates durable complete regressions in some patients with metastatic melanoma [45]. One promising strategy to boost the efficiency of ACT is to administer T cells that have been genetically engineered to express a T-cell receptor (TCR) that recognizes a specific tumor antigen or a chimeric antigen receptor (CAR) that has been artificially constructed to contain the tumor antigen-binding domain of an antibody fused with a T-cell signaling domain [46]. The success of these therapies partially depends on the expression of a specific and immunogenic antigen in cancer cells that functions as a target for the engineered T cell. The majority of to-date targeted tumor antigens are self-antigens, normally expressed during development and aberrantly expressed by tumors. In this regard, inhibitors of repressive chromatin factors have been proven to induce the expression of some of these antigens. DNA hypomethylating agents induce expression of the antigen NY-ESO-1 in colorectal cancer cells improving the efficacy adoptive T cell therapy against this antigen [47]. Similarly, HDAC inhibitors cause the upregulation of the antigen NKG2DL in ovarian cancer cell lines facilitating their immune recognition by chimeric NKG2D CAR T cells [48].

Importantly, infused T cells must be able to resist mechanisms of exhaustion. Recently, Sen et al. [49] characterized enhancer regions involved in the induction of PD-1 expression during exhaustion by comparing the epigenetic landscape of non-exhausted and exhausted T cells. Of relevance, introducing genetic deletions in these enhancers regions dramatically reduced PD-1 expression. Thus, generating antigen-oriented engineered T cells that also contain mutations in the PD-1 enhancer might be a potential strategy to prevent exhaustion of these cells in ACT strategies.

## 6. Modulating Regulatory T cells

Regulatory T cells (Tregs) constitute an additional strategy to prevent autoimmunity by suppressing aberrant immune responses against self-antigens. However, these cells can also suppress anti-tumor immune responses. Tregs mediate their suppressive activity by several mechanisms, which include suppression of antigen-presenting cells via CTLA-4 and secretion of inhibitory cytokines. Infiltration of a large number of Tregs into tumor tissues is often associated with poor prognosis. Accumulating evidence suggests that Treg depletion might be able to reestablish and enhance anti-tumor immune responses [50].

**Figure 2:**
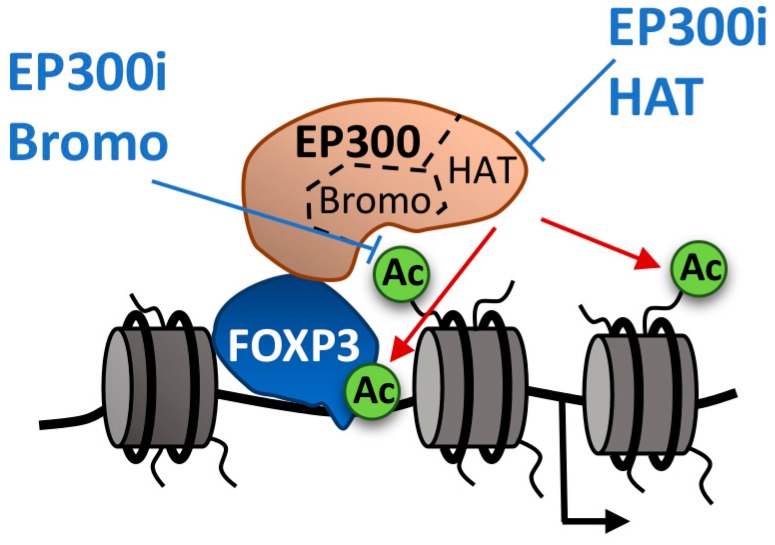
FOXP3 binds to responsive elements in the DNA and recruits histone acetyltransferases, such as EP300 to facilitate transcription. EP300 requires both the binding of the bromodomain to acetylated histones and the HAT domain to acetylate both FOXP3 and histone tails (red arrows) at FOXP3-regulated promoters. Inhibitors of the EP300 bromodomain (blue arrows) impair the proper recruitment of EP300 to chromatin and the acetylation of histones, causing a decrease in gene expression.

Tregs express high levels of the transcription factor FOXP3 that controls the gene expression programs involved in Treg function. The histone acetyltransferase EP300 acetylates FOXP3 increasing its stability by preventing ubiquitin-dependent degradation [51] and it is also likely that it participates as a cofactor in FOXP3-dependent transcription through histone acetylation. Conditional deletion of EP300 in Tregs impairs Treg cell suppressive functions and peripheral Treg cell induction, limiting tumor growth in mice [52]. The EP300 HAT inhibitor C646 [53] recapitulates these effects suggesting that chemical inhibition of EP300 offers a good opportunity to modulate Treg function [53]. However, C646, which is the most potent and selective EP300 HAT inhibitor reported this far, shows low potency in cells, likely due to its affinity for cysteine-rich proteins [54]. More recently, inhibitors of the EP300 bromodomain have been developed. Treatment of Tregs with these inhibitors led to a reduction in FOXP3 expression both at protein and transcript levels, which was coincident with lower levels of FOXP3 acetylation and lower levels of histone acetylation at the FOXP3 locus [14]. The fact that the EP300 bromodomain is required for the proper binding of EP300 to chromatin [12] and that bromodomain inhibitors do not affect EP300 HAT activity [14], suggest that FOXP3 acetylation is likely to take place in the chromatin (Figure 2). FOXP3·has been described to interact with several chromatin-related proteins [55,56] that might be pharmacological candidates to modulate FOXP3 activity and Treg function.

## 7. Rewiring the Interferon Pathway

Interferons are cytokines with a long history of involvement in the development and treatment of cancer. Of the three major types of interferons, type I IFNs (most studied IFNα and IFNβ) can be produced by most cell types in the organisms, while type II (IFNγ) and III IFNs are restricted to specific cell types, such as T cells and natural killer cells (NK) [57]. The binding of interferons to their transmembrane receptors stimulates the activation of associated JAK tyrosine kinases that result in phosphorylation and activation of signal transducers and activators of transcription (STATs) which dimerize and translocate to the nucleus to drive the expression of IFN-stimulated genes. IFNs regulate multiple facets of antitumor biology. They directly affect the immune system by promoting the antitumor activity of T cells, NK cells, and DCs, and inhibiting the activity of immune-suppressive cells, such as Tregs. In addition, IFNs have important effects in tumor cells including inhibiting cell cycle, promoting apoptosis and improving antigen expression and presentation. Eventually, tumors might become resistant to the action of IFNs in several ways including deletion of IFN type I genes and alteration of JAK-STAT components of the IFN-induced signaling [57]. Epigenetic therapies might help to restore the sensitivity of tumors to IFN, or even stimulate IFN production by tumor cells or immune cells in the tumor microenvironment.

**Figure 3:**
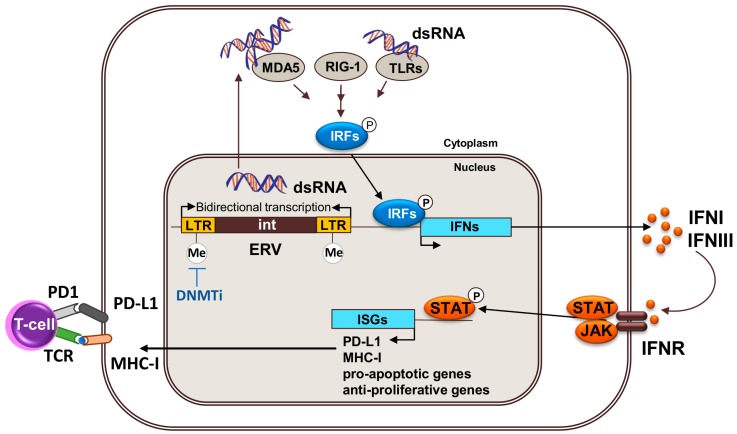
DNMTs inhibitors activate the expression of endogenous retroviruses in cancer cells. Treatment with DNMTs inhibitors (blue arrow) causes loss of DNA methylation in the long terminal repeats (LTR) of endogenous retroviruses (ERVs) and activation of bidirectional transcription, which leads to the formation of double-stranded RNA (dsRNA). dsRNA are sensed in the cytoplasm by cytosolic receptors MDA5/RIG-I/LTRs that initiate signaling cascades to mediate the phosphorylation of interferon responsive factors (IRFs) that, in turn, activate the expression of target genes in the nucleus, including IFNs. INFs activate JAK/STAT-dependent signaling pathways, which result in the induction of interferon stimulated genes (ISGs).

The molecular mechanism by which DNA methyltransferases inhibitors show clinical efficacy is of intense debate and several mechanisms have been proposed (as discussed above). The most accepted mechanism is that it reactivates the expression of tumor suppressors by reversing the abnormal DNA methylation in their promoters. However, the prolonged time to response observed in patients, and the lack of a global DNA methylation profiling predictive signature for response, suggests the existence of other mechanisms beyond the demethylation of tumor suppressor’s promoters. In NSCLC, breast, colorectal, and ovarian cancer cell lines, AZA treatment upregulates genes and pathways related to IFN and immunity [27,28]. Two recent papers show that AZA stimulates these IFN-related signatures through the induction of endogenous retroviruses (ERVs) that are silenced through DNA methylation in cancer cells [26,39]. Loss of DNA methylation correlates with re-expression of ERVs through bidirectional transcription that results in the production of double stranded RNAs (dsRNA). These dsRNAs are sensed by viral defense proteins that, in turn, stimulate the production of IFN and the activation of anti-viral, anti-proliferation, and pro-apoptotic pathways in cancer cells (Figure 3) [26,39]. Furthermore, the production of IFN might contribute to facilitate the infiltration and activation of T cells into the tumor microenvironment, stimulate tumor antigen presentation to T cells by dendritic cells and have a negative impact on Treg proliferation [57]. In addition to PD-L1 re-expression, the stimulation of IFN production in cancer cells through re-activation of endogenous retroviruses provides an alternative mechanisms for the improved therapeutic efficacy of anti-PD-L1 or anti-CTLA4 and AZA combinations [25,26] in which anti-PD-L1 or anti-CTLA4 therapy contributes to alleviate T cell exhaustion caused by prolonged exposure to AZA-induced IFN.

Importantly, the reactivation of dsRNAs and concomitant immunological signatures hold unprecedented predictive value for patient response. Comparison of the basal expression of AZA-induced IFN-related genes within a collection of ovarian, breast, colon, and lung cancers reveals the existence of low- and high-expressing groups of patients [26]. Interestingly, highly-expressing subtypes appear more sensitive to immune checkpoint therapy, raising the possibility that treatment of patients with a low expression signature with AZA improves responses to immune checkpoint inhibition. Moreover, monitoring the re-expression of dsRNA might be a better predictor of patient response than monitoring DNA methylation levels [39].

## 8. Conclusions and Future Directions

Increasing evidence suggests that epigenetic drugs can improve the therapeutic responses to cancer immunotherapies. As reviewed here, immunotherapies might also contribute to palliate acquired resistance to the treatment with epigenetic drugs. Perhaps the most attractive potential is the fact that several epigenetic inhibitors can block the proliferation and viability of cancer cells while potentiating the actions of the immune system. However, the inhibition of epigenetic regulators has pleiotropic effects that affect multiple pathways both in cancer cells and immune cells and, therefore, dissecting the molecular mechanisms involved in the responses is not straightforward. In addition to DNMT and HDAC inhibitors, which are the epigenetic drugs with longer therapeutic history, inhibitors of other classes of epigenetic regulators are emerging and might show immunomodulatory properties.

## Figures and Tables

**Table 1:** Described effects of inhibitors of the indicated epigenetic targets and targeted domains in immuno-oncology-related pathways.

Target	Domain	Immuno-Modulatory Effects
DNMTs	Methyltransferase	Upregulation of PD-L1/PD-L2 in cancer cells
		Upregulation of cancer testis antigens (CTAs)
		Upregulation of genes of the MHC presentation pathway
		Reactivation of repetitive elements (including ERVs)
HDACs	Deacetylase	Upregulation of PD-L1/PD-L2 in cancer cells
		Upregulation of cancer testis antigens (CTAs)
		Upregulation of genes of the MHC presentation pathway
		Reactivation of repetitive elements
		Upregulation of T cell chemokine expression
BRD4	Bromodomain	Downregulation of PD-L1 in cancer cells
EP300	Acetyltransferase Bromodomain	Downregulation of FOXP3 in Tregs
